# Psychological perspectives on divine forgiveness: seeking divine forgiveness

**DOI:** 10.3389/fpsyg.2024.1256402

**Published:** 2024-02-21

**Authors:** Frank D. Fincham, Heather M. Maranges

**Affiliations:** Human Development and Family Science, Florida State University, Tallahassee, FL, United States

**Keywords:** divine forgiveness, God image, God attachment, religiosity, resilience

## Abstract

Seeking divine forgiveness (forgiveness by a Supreme Being or Higher Power) is important because the perception of such forgiveness is associated with psychological well-being This paper is the first to examine a process model of divine forgiveness in which the decision to pursue such forgiveness initiates the process of seeking it. Two studies investigate the likelihood of seeking divine forgiveness. Study 1 (*N* = 190) introduces and provides discriminant validity for a unidimensional measure divine forgiveness seeking. Convergent validity is provided by demonstrating that seeking divine forgiveness correlates with reported experiences of divine forgiveness both concurrently and six weeks later. Study 2 (*N* = 390) provides a confirmatory factor analysis of seeking divine forgiveness scale items identified in Study 1 and replicates the concurrent and temporal association with reported experiences of divine forgiveness using a longer time interval (12 weeks). It also documents associations between a person’s image of God, attachment and closeness to God and the likelihood of seeking divine forgiveness. Both studies control for religiosity and Study 2 introduces an additional control for impression management. Together, they provide support for the idea that the decision to pursue divine forgiveness begins the process of seeking such forgiveness. We discuss limitations of the research and outline several paths for additional studies.

## Introduction

Divine forgiveness (the forgiveness of a Higher Power or Supreme Being) is found in many religions (Lundberg, 2010), and most of the world’s population professes a religious faith (Pew Research Center, 2012). Based on those twin observations and building on a review and analysis of empirical research on divine forgiveness (Fincham, 2022), Fincham and May (2023a) outline a psychological model of the processes involved in the human quest for divine forgiveness. Central to this model are several decision points. The first involves whether to seek divine forgiveness. Seeking divine forgiveness is important because the perception of such forgiveness is associated with psychological well-being both concurrently (e.g., Fincham and May, 2022a; Kim et al., 2022) and over time (Long et al., 2020). Given the rudimentary nature of research on divine forgiveness (see Fincham, 2022 for a review), it is perhaps not surprising that there are no data on the likelihood of seeking divine forgiveness. The present study seeks to redress this issue and in doing so provides the first test of the Seeking-Experiencing Divine Forgiveness Model (Fincham and May, 2023a).

### Initiating the quest for divine forgiveness: theory

Forgiveness is a potential response to a wrong. The wrong may be a transgression against God or another person and can be experienced as a sin. In the prodigious forgiveness literature on human forgiveness, the wrong typically comprises the stated thoughts (words) or overt actions (deeds) of a person (see Worthington and Wade, 2020). Humans may also seek divine forgiveness for what they have said or done and may even see their behavior as a moral transgression that is sinful. However, divine forgiveness differs from human forgiveness in that there is a third type of wrong for which it may be sought. In addition to words and deeds, a person might seek divine forgiveness for thoughts that they may perceive as sinful, wrong, evil, or hurtful. For example, Christianity speaks of sin in the heart (e.g., Mathew, 5:28) and Islam also refers to “qalbin salîm” or a pure heart. Consequently, Fincham and May (2023a) include wrongful thoughts, words and deeds as potential sources that may give rise to seeking divine forgiveness. Nonetheless, it needs to be pointed out that the status of mental events as morally consequential varies depending on the religion (Cohen and Rozin, 2001) and thus whether thoughts as wrongs require divine forgiveness is not universal.

To be worthy of forgiveness, the thought, word, or deed must be perceived as a wrong by the agent experiencing it. Typically, such perceptions arise in regard to what Heider (1958) called an “ought” (“what ought to be done or experienced, independent of the individual’s wishes,” p. 219). When a wrong is perceived, the perpetrator of the wrong may choose to seek divine forgiveness. Possibly the most obvious factor in this decision is religious/spiritual beliefs. An atheist is not likely to pursue divine forgiveness. But a wrongdoer who is a theist may not seek divine forgiveness. Whether such an individual seeks divine forgiveness most likely depends on their perception of God or God image. A substantial body of research (see Sharp et al., 2021 for an overview) has revealed one view of God as benevolent/kindly (e.g., “forgiving,” “loving”) and one of an authoritarian/wrathful God (e.g., “punishing,” “critical”). When God is seen primarily as authoritarian or wrathful, the person is unlikely to seek God’s forgiveness, whereas a person who views God primarily as benevolent or kind or benevolent is more likely to seek forgiveness.

The person’s relationship to God, may also influence whether they embark on the quest for divine forgiveness. Here, closeness and attachment to God likely matter. Anxiously attached individuals who are preoccupied with the availability of their attachment figure (God in this case) in all likelihood seek divine forgiveness. These people have difficulty managing negative emotions they experience and expect their distress to be contained by significant others (Shaver and Mikulincer, 2002). Conversely, avoidant attachment may negatively predict the likelihood of seeking divine forgiveness, given that individuals high in avoidant attachment tend to avoid disclosing personal details and do not rely on close others to manage their feelings (Shaver and Mikulincer, 2002). As regards closeness to God, it seems reasonable to argue that those who feel closer to God as compared to those who feel more distant, are more likely to seek divine forgiveness. Indeed, transgressors tend to be prompted to seek human forgiveness when the transgressor and victim are closer, the transgression is more severe, and the person ruminates about it (Riek, 2010).

By showing that the likelihood of seeking divine forgiveness is related to factors such as the person’s attachment to God and their closeness to God, the present investigation has the potential to make an important contribution. This is because it would demonstrate that the same constructs that influence interpersonal forgiveness may operate in divine forgiveness. Showing such a correspondence would suggest that there is no need to posit mechanisms beyond those found in the understanding of forgiveness between humans to understand divine forgiveness.

### Initiating the quest for divine forgiveness: pragmatics

Human and divine forgiveness are different (Root-Luna et al., 2023), but as evidenced in a recent analysis of research on divine forgiveness, the literature on human forgiveness has been a useful resource in guiding the development of an agenda for systematic research on divine forgiveness (Fincham, 2022). However, when it comes to seeking forgiveness, research on human forgiveness is less helpful as this topic has received little attention compared to the granting of forgiveness. Thus, for example, there are no measures on the likelihood of seeking forgiveness even though there is some research on the reciprocal relationships among seeking forgiveness, receiving forgiveness, and self-forgiveness (e.g., Wenzel et al., 2021), on some elements of forgiveness seeking such as apologies and restitution (e.g., Hornsey et al., 2020; Forster et al., 2021), and on physiological correlates of forgiveness-seeking imagery (e.g., da Silva et al., 2017; Witvliet et al., 2020).

In light of these observations, the current attempt to understand the first decision point in Fincham and May’s (2023a) component analysis, understanding the likelihood of seeking divine forgiveness must begin *ab initio*. The starting point is self-evident, a psychometric exercise that entails development of a scale to measure the likelihood of seeking divine forgiveness. Notwithstanding the earlier observation that the human forgiveness literature provides little guidance on forgiveness seeking, we begin there. There are 45 measures of human forgiveness (Fernández-Capo et al., 2017) and they were examined to determine whether any might lend themselves to adaptation for assessing the *likelihood of seeking* divine forgiveness. One such measure was identified, the Transgression Narrative Test of Forgivingness (Berry et al., 2001). This measure comprises five scenarios each depicting a transgression. After each scenario respondents indicate the likelihood of forgiving the transgressor. Adaptation of this measure therefore served as the starting point for the current research.

### Current research

The present paper is the first to examine the Seeking-Experiencing Divine Forgiveness Model (Fincham and May, 2023a). It reports two studies that investigate an initial component of this recently proposed process model of seeking divine forgiveness. This initial component of the model is critical as it initiates the process of seeking divine forgiveness, the receipt of which is, as noted earlier, related to psychological well-being both concurrently and longitudinally. The first study investigates the viability of using an adapted version of the Transgression Narrative Test of Forgivingness to assess the likelihood of seeking divine forgiveness. It does so by using an exploratory factor analysis and examines whether the resulting scale predicts concurrent and later reported experiences of divine forgiveness. The second study follows up with a confirmatory factor analysis, replicates whether seeking divine forgiveness predicts concurrent and later experiences of divine forgiveness and goes on to examine potential predictors of the individuals’ likelihood of seeking divine forgiveness. Specifically, it examines whether a person’s image of God, attachment, and closeness to God predict the likelihood of seeking divine forgiveness. Documenting factors that predict the seeking of divine forgiveness is important as they might potentially serve as points of intervention in attempts to increase the likelihood of seeking and consequently receiving divine forgiveness.

## Study 1

This study creates and assesses the validity of a divine forgiveness seeking measure. It does so by adapting one of the most sophisticated instruments in the literature on human forgiveness. Specifically, the Transgression Narrative Test of Forgivingness (TNTF) was developed using a Rasch rating scale model and is designed to provide a measure of the likelihood of forgiving transgressions portrayed in five narratives that reflect variation in protagonists (relative, friend, acquaintance) and the nature of the transgression (intentional act, negligent act). It was anticipated that the adapted TNTF would yield a unidimensional measure. In support of its construct validity, we expected that the measure would be positively related to concurrent reports of experienced divine forgiveness and to predict later reports of experienced divine forgiveness. To rule out the alternative explanation that relations involving the likelihood of divine forgiveness and its correlates may simply reflect religiosity, we statistically control for level of religiosity. To examine discriminant validity, two variables theoretically unrelated to the likelihood of seeking divine forgiveness were assessed: emotion regulation and resilience.

### Method

#### Participants and procedure

Undergraduate students (*N* = 190) at a large Southeastern public university were participants. Registered in social studies and human science courses, most were women. Only those who indicated their belief in “supernatural agents(s) (e.g., God, Gods, a higher power)” were eligible for the study. Of the 190 participants, 173 (91%) were women, with 132 (69.5%) identifying as White, 18 (9.5%) as Black, 22 (11.6%) as Latina/o, 6 (3.2%) as Asian, 8 (4.2%) as mixed race, 1 as Native American, 2 as Native Hawaiian/Pacific Islander, and 1 declined to provide ethnic/racial information. The average age was 19.81 (SD = 1.24) years.

Students were recruited from social studies and human science courses early in the semester by giving them the chance to respond to 2 online surveys separated by six weeks as one of several options to gain some extra credit. The local Institution Review Board approved all materials and procedures before the study was conducted.

### Measures

#### Likelihood of seeking divine forgiveness

As noted, TNTF scenarios were adapted. Below is an example with the adaptation shown in parentheses.

“A friend offers (You offer) to drop off a job application for you (for a friend) at the post office by the deadline for submission. A week later, you get (your friend gets) a letter from the potential employer saying that your (his/her) application could not be considered because it was postmarked after the deadline, and they had a very strict policy about this. Your friend said that he or she met an old friend (You remember that you met an old friend), went to lunch, and lost track of time. When he or she (When you) remembered the package, it was close to closing time at the post office and he or she (you) would have to have rushed frantically to get there; he or she (you) decided that deadlines usually aren’t that strictly enforced so he or she (you) waited until the next morning to deliver the package. Imagine yourself in such a situation and mark how likely you are to forgive your friend for not delivering the application on time (Imagine yourself in such a situation and indicate how likely you are to seek God’s forgiveness for not delivering the application on time.)”

One of the scenarios did not lend itself to adaptation and consequently a new scenario was generated. As there was uncertainty about the viability of all the adapted scenarios a second new scenario was also generated. After each scenario, respondents indicated how likely they were to seek God’s forgiveness by using a slider with the endpoints labelled 0 and 100. There was a box next to the slider that showed the exact numerical value as they positioned the slider. Scores were summed with higher scores indicating a great likelihood of seeking divine forgiveness.

#### Religiosity

At time 1, participants responded to two commonly used items assessing religiosity (Pearce et al., 2017). They first asked, “How important is religion in your life?” Participants indicated their answers on an 8-point scale from “not at all” to “extremely important.” The second question asked, “How often do you attend religious services or meetings?” Again, participants indicated their answers on an 8-point scale ranging from 0 (“never”) to 7 (“about once a day”). The items correlated strongly (*r* = 0.62) and were therefore summed providing a measure of religiosity (*α* = 0.74).

#### Divine forgiveness

Divine forgiveness was assessed with the 5-item scale used by Fincham and May (2022b, 2023b) and consisted of the following questions “How often have you felt that God forgives you?”; “Knowing that I am forgiven for my sins gives me the strength to face my faults and be a better person”; “I am certain that God forgives me when I seek His forgiveness”; “How often do you experience situations in which you have the feeling that God delivers you from a debt?” “How often do you experience situations in which you have the feeling that God is merciful to you?” For the first 3 items participants indicated their responses on a 4-point scale and for the remaining items a 5-point scale was used. In the present sample, reliability was satisfactory (α = 0.86 at Time 1 and 0.90 at Time 2).

#### Emotion regulation

Individual difficulty in regulating emotions was assessed with the Difficulties in Emotion Regulation Scale (DERS; Gratz and Roemer, 2004; Victor and Klonsky, 2016). The 18-item measure was administered (Victor and Klonsky, 2016). In the present study sample reliability was satisfactory (*α* = 0.89). Some example items are, “when I’m upset, I lose control over my behaviors,” “when I’m upset, I have difficulty concentrating,” and “when I’m upset, I feel guilty for feeling that way.” Responses were given on a scale of 1 (“almost never”) to 5 (“almost always”) with higher summed scores showing greater difficulties in regulating emotions.

#### Resilience

According to Smith et al. (2008, p. 194), “The brief resilience scale (BRS) was created to assess the ability to bounce back or recover from stress.” It comprises 6 items (e.g., “I tend to bounce back quickly after hard times”; “I tend to take a long time to get over setbacks in my life” – reverse scored) answered on a 5-point scale ranging from 1 (“strongly disagree”) to 5 (“strongly agree”). In the present study sample reliability was satisfactory (α = 0.80). Higher summed scores indicate greater resilience.

### Results and discussion

#### Factor analysis

An exploratory factor analysis (principal axis factoring and varimax rotation) using SPSS was used to examine the six items that assessed the likelihood of seeking divine forgiveness. The analysis yielded a single factor with an eigenvalue of 4.93 that accounted for 73.21 percent of the variance. However, one item loaded lower than all the others on the factor. Closer inspection showed that responses on this item clustered at the high end of the response scale (over 50% of responses fell in the top fifth of the response scale with 24.1% comprising the maximum value). In addition, it was found that coefficient alpha would increase if this item were not included in the measure. The factor analysis was therefore repeated without this item and again yielded a single factor with an eigenvalue of 3.82 accounting for 76.38 percent of the variance (see Table 1). Coefficient alpha for the five-item scale was 0.94. This measure was used in subsequent analyses.

**Table 1:** Narrative test of seeking divine forgiveness items and their factor loadings.

Item	Loading
1. You have a paper due at the end of the week when you run into someone who you occasionally see in the class. This person has already completed the paper for the class, and you tell them that you feel under a lot of time pressure and ask them to lend you their paper for some ideas. They agree, and feeling under great duress you find yourself simply retyping the paper and handing it in. The professor recognizes the paper, calls both of you to her office, scolds the person who gave you the paper, and says they are lucky she does not put both of you on academic probation. Imagine yourself in such a situation and mark how likely you are to seek God’s forgiveness for what you did?	0.82
2. You offer to drop off a job application for a friend at the post office by the deadline for submission. A week later, your friend gets a letter from the potential employer saying that his/her application could not be considered because it was postmarked after the deadline, and they had a very strict policy about this. You remember that on the way to the post office you had met an old friend, went to lunch, and lost track of time. When you remembered the package, it was close to closing time at the post office and you would have to have rushed frantically to get there; you had then decided that deadlines usually aren’t that strictly enforced so you waited until the next morning to deliver the package. Imagine yourself in such a situation and indicate how likely you are to seek God’s forgiveness for not delivering the application on time?	0.88
3. You just started a new job and it turns out that a classmate from high school works there, too. Even though the classmate wasn’t part of your crowd, there’s at least a face you recognize. You two hit it off right away and talk about old times. A few weeks later, you are having lunch in the cafeteria with several of your coworkers and tell them about something your old classmate did back in school; they laugh at the story and one co-worker even makes a snide and hostile comment about your old classmate. The next day your old classmate tells you that she was nearby and overheard the conversation with the co-workers and that what you told them is something that she is deeply ashamed of and did not want anyone to know about. Imagine yourself in such a situation and mark how likely you are to ask seek God’s forgiveness for telling others your old classmate’s secret?	0.96
4. An acquaintance tells you about a job that he or she really hopes to be hired for. Without telling them, you then apply for the job and end up getting it. A couple of days later, the acquaintance tells you that they did not get the job and now will not be able to pay their rent. Imagine yourself in such a situation and mark how likely you are to seek God’s forgiveness for what you did?	0.84
5. You accompany your family to a New Year’s party. The atmosphere at the party is warm and friendly. You have a drink and are soon chatting with a group. You end up humiliating a family member by sharing a story about them that they did want anyone outside the family to know. The group laughs at the story; the family member turns red with embarrassment and then leaves the party. Imagine yourself in such a situation and mark how likely you are to seek God’s forgiveness for hurting the family member?	0.87

#### Convergent and discriminant validity

Table 2 contains the means, standard deviations, and bivariate correlations among the study variables. As regards convergent validity, the likelihood of seeking divine forgiveness was related to reports of divine forgiveness both concurrently and six weeks later. Both variables were also strongly related to religiosity, emphasizing the need to rule it out as being responsible for any association between seeking divine forgiveness and reports of experiencing divine forgiveness. When partialled out of the concurrent correlation between them, the association between seeking divine forgiveness and reports of experiencing divine forgiveness was reduced but remained statistically significant, *r* = 0.16, *p* = 0.025. Turning to discriminant validity, the likelihood of seeking divine forgiveness was unrelated to difficulties in emotion regulation and resilience.

**Table 2:** Means, standard deviations (SD) and correlations among study 1 variables.

	1	2	3	4	5	6
1. Seek DF (T1)		0.46	0.50	0.56	0.04	0.00
2. Reported DF (T1)			0.77	0.63	0.21	0.20
3. Reported DF (T2)				0.58	0.12	0.08
4. Religiosity (T1)					0.06	0.10
5. Resilience (T1)						0.48
6. Emotion regulation (T1)						
Mean	298.02	16.06	16.4	3.56	3.98	2.31
Standard deviation	136.29	3.41	3.51	3.58	3.99	0.64

Correlations > 0.14, *p* < 0.05; correlations > 0.18, *p* < 0.01; correlations > 0.23, *p* < 0.001.

To examine the association between likelihood of seeking divine forgiveness and reports of experiencing divine forgiveness six weeks later, we conducted a linear regression analysis. Time 2 reports of divine forgiveness served as the dependent variable with the likelihood of seeking divine forgiveness, Time 1 reports of experiencing divine forgiveness, and religiosity serving as predictor variables. The predictor variables accounted for 63 percent of the variance in the outcome variable. Both likelihood of seeking divine forgiveness, *β* = 0.16, *p* = 0.004, and earlier reports of experiencing divine forgiveness, *β* = 0.65, *p < 0*.001, independently accounted for variance in later reports of experiencing divine forgiveness.

The present findings provide initial evidence on the viability of a measure of the likelihood of seeking divine forgiveness. This novel measure correlated with concurrent reports of divine forgiveness and predicted reports of divine forgiveness six weeks later. This is notable given the high correlation of 0.77 between the two reports of experiencing divine forgiveness and that we controlled for religiosity as well. This is to say that the more likely people are to seek divine forgiveness when presented with cases of their transgressions, the more they report experiencing God’s forgiveness concurrently and six weeks later. This is not due to individuals’ levels of religiosity.

Whether the items identified in this study yield positive results in a confirmatory context, however, is open to question. The next study addresses this concern as well as expands our understanding of how divine forgiveness seeking relates to perceptions of and closeness to a higher power.

## Study 2

In addition to providing a confirmatory factor analysis of the seeking divine forgiveness items identified in Study 1, the present study also replicates and extends the earlier findings by examining whether the likelihood of seeking divine forgiveness predicts reports of divine forgiveness over a longer time period—12 weeks. This ensures that findings in Study 1 are not merely an artifact of the shorter time interval chosen. The study also examines the association between a person’s image of God, attachment, and closeness to God and the likelihood of seeking divine forgiveness. Finally, we also control for socially desirable responding.

### Method

#### Participants and procedure

Undergraduate students (*N* = 390; 354 Women) at a large Southeastern public university were study participants. Again, they were mostly from social and human sciences, where most students are women. Students who indicated that they believed in “supernatural agents(s) (e.g., God, Gods, a higher power)” were included in the sample. Of the 390 participants, 263 (67.4%) identified as White, 39 (10%) as Black, 55 (14.1%) as Latino/a, 8 (2.1%) as Asian, 18 (4.6%) as mixed race, 2 as Native American, 1 as Native Hawaiian/Pacific Islander, and 2 chose not to respond to the question. The participants average age was 19.91 (SD = 1.86) years.

Students were recruited from classes at the beginning of the semester by giving them the chance to respond to 2 online surveys separated by 12 weeks as one of several options to gain some extra credit. The local Institution Review Board had approved all materials and procedures before the study began.

### Measures

The same measures of likelihood of seeking divine forgiveness, reports of experiencing divine forgiveness, and religiosity used in Study1 were again utilized in the present study. Seeking divine forgiveness and reports of divine forgiveness were assessed twice, at an initial assessment (T1) and 12 weeks later (T2). All other measures were obtained at the initial assessment (T1).

#### God image

Mental representation of God or God image was assessed using the A/B-God scale (Johnson et al., 2015) a measure comprising 18 adjectives reflecting an authoritarian God (A-God; e.g., “critical,” “punishing,” “stern”; coefficient alpha = 0.94 in the present sample) and a benevolent God (B-God; e.g., “caring,” “loving,” “merciful”; coefficient alpha = 0.89 in the present sample). However, the adjective “forgiveness” in the B-God portion of the scale was not used as doing so would introduce a level of tautology given that it is being used to predict likelihood of seeking divine forgiveness.

#### Attachment to god

The Attachment to God Scale (Rowatt and Kirkpatrick, 2002) comprises 9 items that assess anxious attachment to God (3 items; e.g., “God sometimes seems very warm and other times very cold to me”) and avoidant attachment (6 items; e.g., “God seems to have little or no interest in my affairs,” “God knows when I need support” – reverse coded). Each item was answered on a seven-point scale (1 = “not true,” 7 = “very true”). Items were summed, and higher scores showed greater anxious attachment and avoidant attachment.

#### Closeness to god

Closeness to God was measured using an adaptation of the widely used Inclusion of Other Scale that assesses closeness in interpersonal relationships (Aron et al., 1992). This single item scale consists of seven pairs of circles that are arranged from no overlap (1, low closeness) and then vary in degree of overlap to nearly overlapped (7, high closeness). Within each circle the label of “self” or “other” appears. In the present study God was substituted for other. Participants are instructed to pick the number from 1 to 7 that best illustrates their relationship with God.

#### Impression management

Impression management was assessed using 8-items from the Balanced Inventory of Desirable Responding Short Form (Hart et al., 2015). This impression management subscale consists of questions indicating “a conscious dissimulation of responses to create a socially desirable image” (Hart et al., 2015, p. 2). It is strongly related to the longer Marlowe-Crowne Social Desirability Scale (*r* = 0.53). Some example items are “I never cover up my mistakes” and “I sometimes tell lies if I have to” (reverse scored). A 7-point response scale was used that went from “strongly disagree” to “strongly agree” with the midpoint labelled “neither agree nor disagree.” Coefficient alpha in the present sample was 0.65. Summed scores show that higher scores represent greater impression management.

### Results and discussion

#### Factor analysis

A confirmatory factor analysis was conducted using the five items of the Narrative Test of Seeking Divine Forgiveness. This analysis was conducted using MPlus. The following criteria are conventionally used to evaluate acceptable model fit: (a) comparative fit index (CFI) that is be greater than 0.95; (b) root mean square error of approximation (RMSEA) less than 0.08, and (c) a standardized root mean square residual (SRMR) lower than 0.08. The fit indices for the specified confirmatory model, with a single underlying factor, showed that the model was a good fit to the data, χ^2^ = 12.091, *p* = 0.03, CFI = 0.995, RMSEA = 0.059, SRMR = 0.011.

#### Primary analyses

Table 3 contains the means, standard deviations, and bivariate correlations among the study variables. Again, seeking divine forgiveness and reported divine forgiveness were strongly correlated both concurrently and over time, i.e., 12 weeks later. That is, T1 seeking divine forgiveness was associated with both T1 reported divine forgiveness and T2 reported divine forgiveness. Additionally, T1 seeking divine forgiveness correlated with T2 seeking divine forgiveness, and T2 seeking divine forgiveness was positively related to T2 reports of divine forgiveness. Moreover, having a benevolent image of God and feeling close to God were strongly and positively related to the likelihood of seeking divine forgiveness (all T1). Also as anticipated, avoidant attachment to God was inversely related to the likelihood of seeking divine forgiveness (both T1). These results suggest that the more people view God as benevolent and relationally close to them and the less their relationship with God is characterized by avoidance, the more likely they are to ask for divine forgiveness. We next conducted analyses that allow for interpretation of temporal effects above and beyond control measures.

**Table 3:** Means, standard deviations (SD) and correlations among study 2 variables.

	1	2	3	4	5	6	7	8	9	10	11
Seek DF		0.50	−0.10	0.58	−0.21	−0.71	0.61	0.60	0.07	0.41	0.54
Reported DF			−0.08	0.40	−0.19	−0.51	0.49	0.54	0.07	0.73	0.42
Authoritarian image				−0.23	0.14	0.17	−0.09	−0.06	−0.10	−0.09	0.03
Benevolent image					−0.19	−0.60	0.45	0.39	0.07	0.22	0.41
Anxious attachment						0.40	−0.21	−0.22	−0.11	−0.26	−0.26
Avoidant attachment							−0.65	−0.61	−0.11	−0.42	−0.57
Closeness								0.63	0.13	0.42	0.46
Religiosity									0.02	0.49	0.53
Impression manage										0.05	0.05
Reported DF (T2)											0.42
Likelihood DF (T2)											
Mean	308.55	16.68	24.12	41.84	9.93	16.16	3.91	7.94	32.11	334.12	17.30
Standard deviation	148.70	3.50	12.09	8.44	4.24	8.33	1.78	3.75	6.48	123.63	3.04

For *r* > 0.10, *p* <. 05; for *r* > 0.18, *p* <0.001.

**Figure 1:**
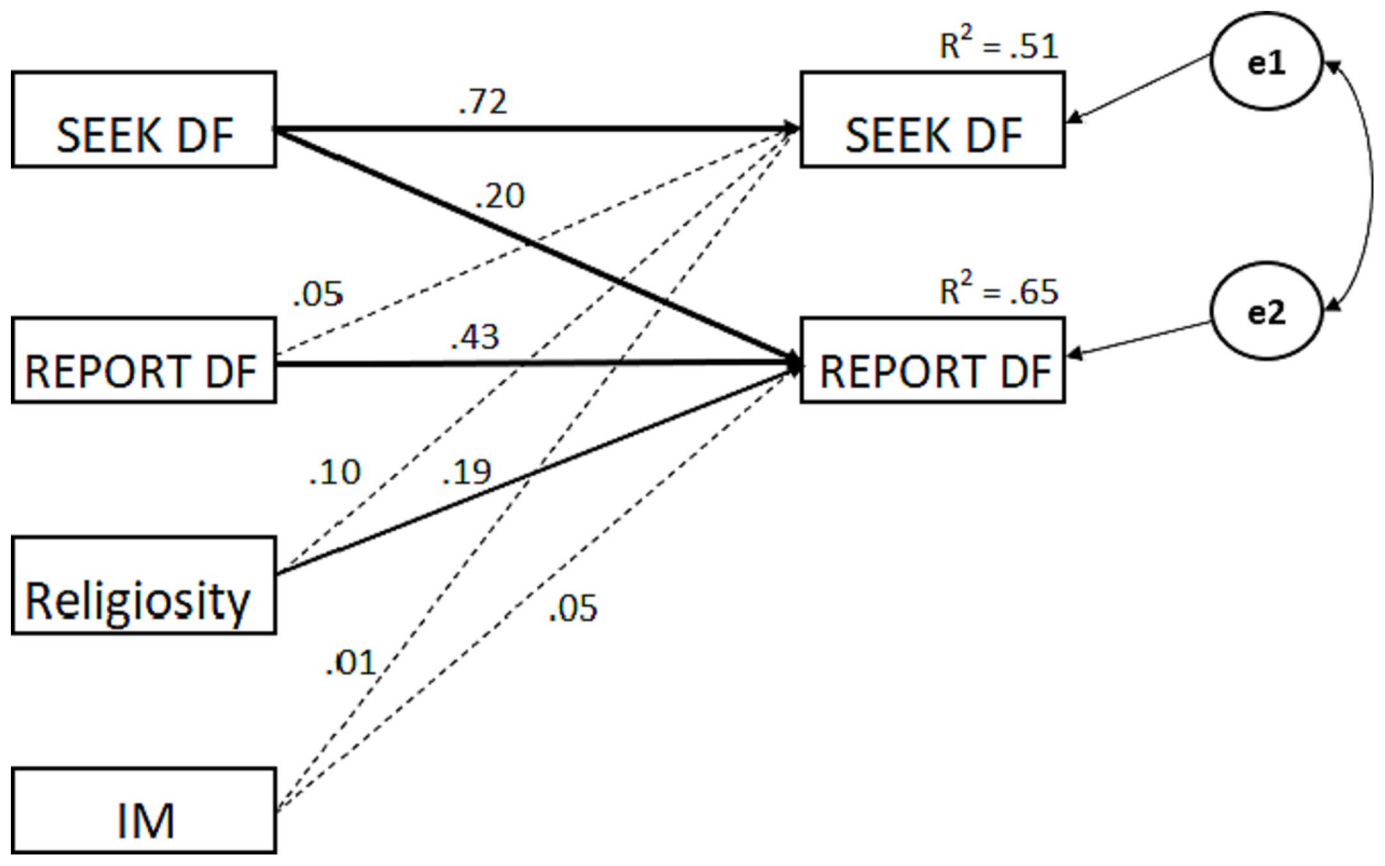
Maximum likelihood estimation of the cross-lagged stability model controlling for religiosity and impression management. DF = divine forgiveness, IM = impression management. Solid lines reflect statistically significant relationships (*p* <. 01).

To examine the longitudinal relations between seeking divine forgiveness and reports of divine forgiveness, we examined a cross-lagged stability model. In this model each Time 2 variable (i.e., seeking divine forgiveness and reported divine forgiveness) was simultaneously regressed on each Time 1 variable (i.e., seeking divine forgiveness and reported divine forgiveness plus controls for religiosity and impression management) using Amos 28. Thus, the stability of each construct is controlled in assessing longitudinal associations. Significant cross-lagged effects show a relationship beyond that which reflects the stability of the constructs and their initial association. The model was fully saturated (no degrees of freedom) and therefore model fit cannot be assessed. Thus, the focus is on parameter estimates. As seen in Figure 1, the cross-lagged coefficient for the path from Time 1 likelihood of seeking divine forgiveness to Time 2 reports of divine forgiveness was significant, *β* = 0.20, *p* < 0.001. In contrast, the cross-lag from Time 1 reports of divine forgiveness to Time 2 likelihood of seeking divine forgiveness was not significant, *β* = 0.05, *p* = 0.34. This provides evidence consistent with the view that seeking divine forgiveness initiates the process of asking for and experiencing God’s forgiveness over time.

The association between a person’s image of God, attachment, and closeness to God and the likelihood of seeking divine forgiveness was examined via linear regression analyses using variables obtained at the initial assessment (T1). The dependent variable was likelihood of seeking divine forgiveness, and God image (authoritarian and benevolent), attachment to God (anxious and avoidant), and closeness to God were independent variables. Both religiosity and impression management were included in the equation as control variables. Before computing the regression equation, the variance inflation factors (VIF) and tolerance values were examined to assess potential multicollinearity. The highest value for VIF was 2.27, which was below the standard cutoff value of 5. Regarding tolerance, the lowest value was 0.44, which was acceptable as only values close to zero (< 0.2) are considered problematic. The equation accounted for 36% of the variance in seeking divine forgiveness, *F* (7, 382) = 30.92, *p* < 0.001. As anticipated, the variables of viewing God as benevolent, *β* = 0.12, *p* = 0.022, avoidant attachment to God, *β* = −0.16, *p* = 0.021, and closeness to God, *β* = 0.13, *p* = 0.027, each accounted for variance in seeking divine forgiveness. Contrary to expectation, neither an authoritarian view of God, *β* = 0.004, *p* = 0.93, nor anxious attachment to God, *β* = 0.01, *p* = 0.82 accounted for unique variance in seeking divine forgiveness. Finally, religiosity, *β* = 0.311, *p < 0*.001, was a significant control variable. The implication is that seeking divine forgiveness is more likely for individuals who view God as benevolent, close, and in a low avoidance relationship with them—providing both theory- and convergent validity-relevant evidence for the construct network of seeking divine forgiveness.

## General discussion

The present research is the first to examine a new process model of divine forgiveness. Fundamental to this model is the decision to seek divine forgiveness after perceived wrongdoing. In combination, the studies provide evidence to suggest that the likelihood of seeking forgiveness can be reliably measured. Study 1 showed that the likelihood of seeking divine forgiveness was strongly related to concurrent reports of receiving divine forgiveness and to reports of divine forgiveness obtained 6 weeks later. As regards discriminant validity, this study showed that the likelihood of seeking divine forgiveness was unrelated to emotion regulation and resilience as theoretically expected.

Study 2 not only replicated the concurrent relationship with reports of divine forgiveness but also showed that seeking divine forgiveness was related to reported divine forgiveness 12 weeks later, demonstrating that the temporal relationship between the two variables was not an artifact of the time lag used in Study 1. The cross-lagged stability model also showed that reports of divine forgiveness were not significantly related to the later likelihood of seeking divine forgiveness suggesting that the temporal relation between the two variables is not bidirectional. Importantly, Study 2 results emerged above and beyond the contributions of religiosity, which was associated with seeking divine forgiveness, and impression management, which was not associated with seeking divine forgiveness.

The current findings are both novel and of theoretical interest. It is axiomatic that the study of divine forgiveness would not be possible without humans seeking to obtain such forgiveness, something that has not been studied to date. It is therefore relevant to identify what predicts the likelihood of seeking divine forgiveness, an issue pursued in Study 2. That is, we aimed to understand who tends to seek divine forgiveness. The case was made that having a benevolent view of God and that having a close relationship to God would be related to seeking divine forgiveness. This was indeed found to be the case. Contrary to expectation, viewing God as authoritarian was not inversely related to seeking divine forgiveness, a finding that might reflect the fact that in the sample studied views of God were predominantly benign. In future research it will be important to examine samples that represent more strongly authoritarian views of God. It was also argued that attachment to God would predict the likelihood of seeking divine forgiveness. Support for this view emerged as avoidant attachment was inversely related to seeking divine forgiveness. As regards anxious attachment, the negative correlation with the likelihood of seeking divine forgiveness was significant, suggesting that individuals higher (vs. lower) in anxious attachment are less likely to seek divine forgiveness. This may reflect an ironic effect of anxious attachment by which the individual is less likely to engage in ethics-relevant cognition due to a focus on the self (e.g., Maranges et al., 2022). However, when examined in a multivariate context, anxious attachment was unrelated to seeking divine forgiveness. Thus, this research is the first to establish that the person who views God as kind and benevolent, close, and as having a low avoidance relationship with them is more likely to seek God’s forgiveness after engaging in some wrong compared to the person who does not.

The preceding observation regarding anxious attachment emphasizes the need to study additional potential predictors of the likelihood of seeking divine forgiveness. To the extent that anxious attachment is associated with ethical disengagement in relation to other people (Maranges et al., 2022), it may be important to assess people’s ethical disengagement as it relates to God after they perceive they have engaged in some wrongdoing. Moreover, other relationship features have proved to be important in interpersonal forgiveness and might similarly impact the seeking of divine forgiveness (Fincham and Beach, 2013). Chief among these is commitment. It is likely that the relationship found between religiosity and the likelihood of seeking divine forgiveness reflects the fact that the former may serve as a proxy for commitment. This makes it important to investigate commitment as it will allow the role of religiosity to be more accurately assessed in the association between the likelihood of seeking forgiveness and reports of divine forgiveness. Relationship satisfaction has also been shown to be related to interpersonal forgiveness and is also worthy of attention.

Additional potential predictors might include social and religious norms. It is important to raise the question of whether divine forgiveness experienced as a result of following such norms differs from that which is obtained when divine forgiveness is intrinsically sought without regard to such external factors. There is some evidence in the forgiveness literature showing that forgiveness motivated by compliance with an externally-based value system is associated with fewer benefits (e.g., Cox et al., 2012). Might seeking divine forgiveness that stems from similar motivation impact the subsequent experience of divine forgiveness? In examining this issue, the distinction between intrinsic and extrinsic religiosity might be important to consider. Other research suggests that people have different psychological representations of and responses to ethical “ought nots” (proscriptive norms) and “oughts” (prescriptive norms) (Janoff-Bulman et al., 2009). For example, people view proscriptive norms as strict and obligatory, whereas prescriptive norms are viewed as akin to moral extra credit. Thus, future research may benefit from examining whether people are more likely to seek forgiveness for violations of proscriptive versus prescriptive norms. In any event, it is clear that a more complete understanding of what predicts the likelihood of seeking divine forgiveness awaits the study of numerous additional relational and nonrelational variables.

## Limitations and conclusion

Despite the novel findings reported, several limitations need to be considered when interpreting the results of the studies. Perhaps the most obvious is that the samples consisted of mostly young, White, college-attending women who identified as Christian. It is therefore important to replicate the current findings with a sample that is more diverse race, gender, religion, and socioeconomic status. Second, further data are needed to map fully the nomothetic network of the construct of seeking divine forgiveness. Third, the measures of seeking divine forgiveness and of the reported receipt of divine forgiveness ask about general tendencies. An important methodological advance would be to study divine forgiveness in the context of specific transgressions. Do the general tendencies studied thus far predict what actually happens in specific situations where divine forgiveness is appropriate? Such investigation would allow us to determine whether the likelihood of seeking divine forgiveness for a specific transgression is related to reported forgiveness from God. Fincham and May’s (2023a) model outlines several factors that may affect this relation and might prove useful when investigating this issue. This relationship might also be mediated and therefore potential mediators such as self-worth (God forgives people for what I did, but will he forgive me?) need to be investigated. Finally, the effect sizes obtained in the temporal relations studied were relatively small. They therefore need to be re-examined using a measurement model in future research that examines longitudinal relations between seeking divine forgiveness and reports of such forgiveness.

Notwithstanding the limitations noted above, the research reported makes a valuable contribution. The data they provide are the first on a novel concept, the likelihood of seeking divine forgiveness, and the type of person who is likely to seek divine forgiveness. Their contribution is emphasized by the fact that this concept is a principal component of a new, process model of divine forgiveness, the Seeking-Experiencing Divine Forgiveness Model (Fincham and May, 2023a). This model outlines numerous processes and factors relevant to understanding divine forgiveness. With its investigation now begun, further research is needed to judge the ultimate utility of the model.

## Data availability statement

The raw data supporting the conclusions of this article will be made available by the authors, without undue reservation.

## Ethics statement

The studies involving humans were approved by Florida State University Institutional Review Board. The studies were conducted in accordance with the local legislation and institutional requirements. The participants provided their written informed consent to participate in this study.

## Author contributions

FF: Conceptualization, Formal analysis, Writing – original draft. HM: Project administration, Writing – review & editing.
